# Supplementary material for: Investigation and Analysis of Pesticide Residues in Four Common Vegetables and Risk Assessment of Dietary Exposure in Ceramic Capital, China
Source: Molecules. 2022 Oct 4;27(19):6562. doi: 10.3390/molecules27196562 (PMC9571922; doi:10.3390/molecules27196562)
Supplement: Supplementary file 1 [file molecules-27-06562-s001.zip › molecules-1892395-supplementary.pdf]

SUPPORTTING INFORMATION

Investigation and Analysis of Pesticide Residues in Four Common Vegetables and Risk Assessment of Dietary Exposure in Ceramic Capital, China

Xingang Meng[1,2,*], Lu Wang[1], Niao Wang[3], Luting Chen[1], Qian Huang[4]

1.College of Biological and Environmental Engineering, Jingdezhen University, Jingdezhen city 333000, Jiangxi province, 334000 P. R. China.

2. Liupanshui Ecological Environment Monitoring Center, Liupanshui city 553000, Guizhou province, P. R. China.

3. Jingdezhen Nursing School, Jingdezhen city 333000, Jiangxi province, P. R. China

*Correspondence: 15885007970@163.com.

Table S1 Determine pesticide species selection

vegetables	Pesticide species							
Leeks	Methamidophos	Ethoprophos	Sulfotep	Chlorpyrifos-methyl	Chlorpyrifos	Dichlorvos	Pyridaben	Fenethanil
Cabbage	Dichlorvos	Omethoate	Diazinon	Monocrotophos	Chlorpyrifos	Malathion	Methidathion	Fenethanil
Cucumber	Omethoate	Pirimicarb	Metalaxyl	Triazolone	Malathion	Fenitrothion	Pendimethalin	Methidathion
Murphy	Pirimicarb	Triazolone	Malathion	Butachlor	Posfolan-methyl	Bifenthrin	Triazophos	Etoxazole

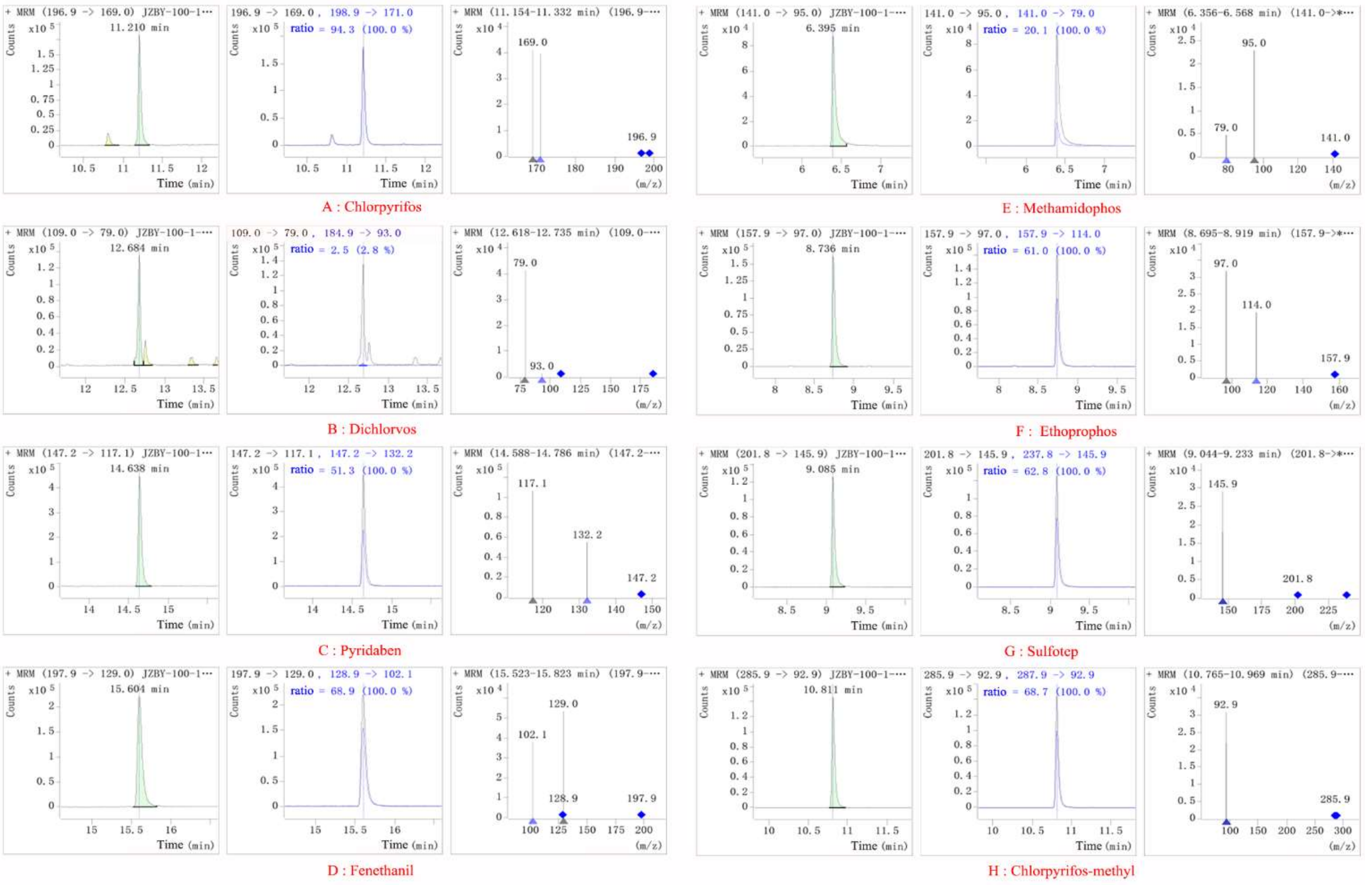

Figure S1 The total ion Chromatogram in MRM Mode, Qualitative and Quantitative Chromatogram of ion pair and Mass Spectrogram (A, Chlorpyrifos; B, Dichlorvos, C, Pyridaben; D, Fenethanil; E, Methamidophos; F, Ethoprophos; G, Sulfotep; H, Chlorpyrifos-methyl).

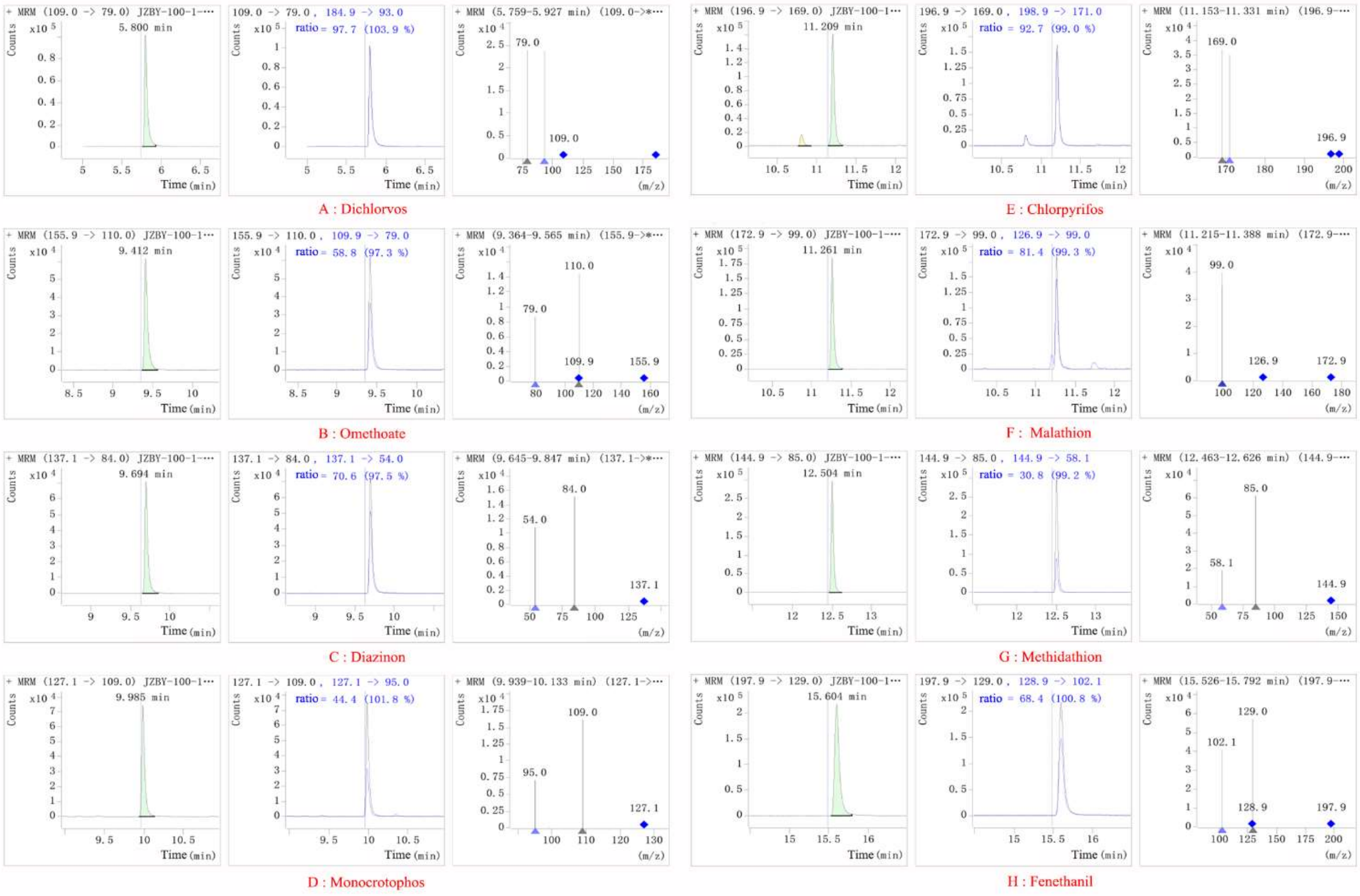

Figure S2 The total ion Chromatogram in MRM Mode, Qualitative and Quantitative Chromatogram of ion pair and Mass Spectrogram (A, Dichlorvos; B, Omethoate, C, Diazinon; D, Monocrotophos; E, Chlorpyrifos; F, Malathion; G, Methidathion; H, Fenethanil).

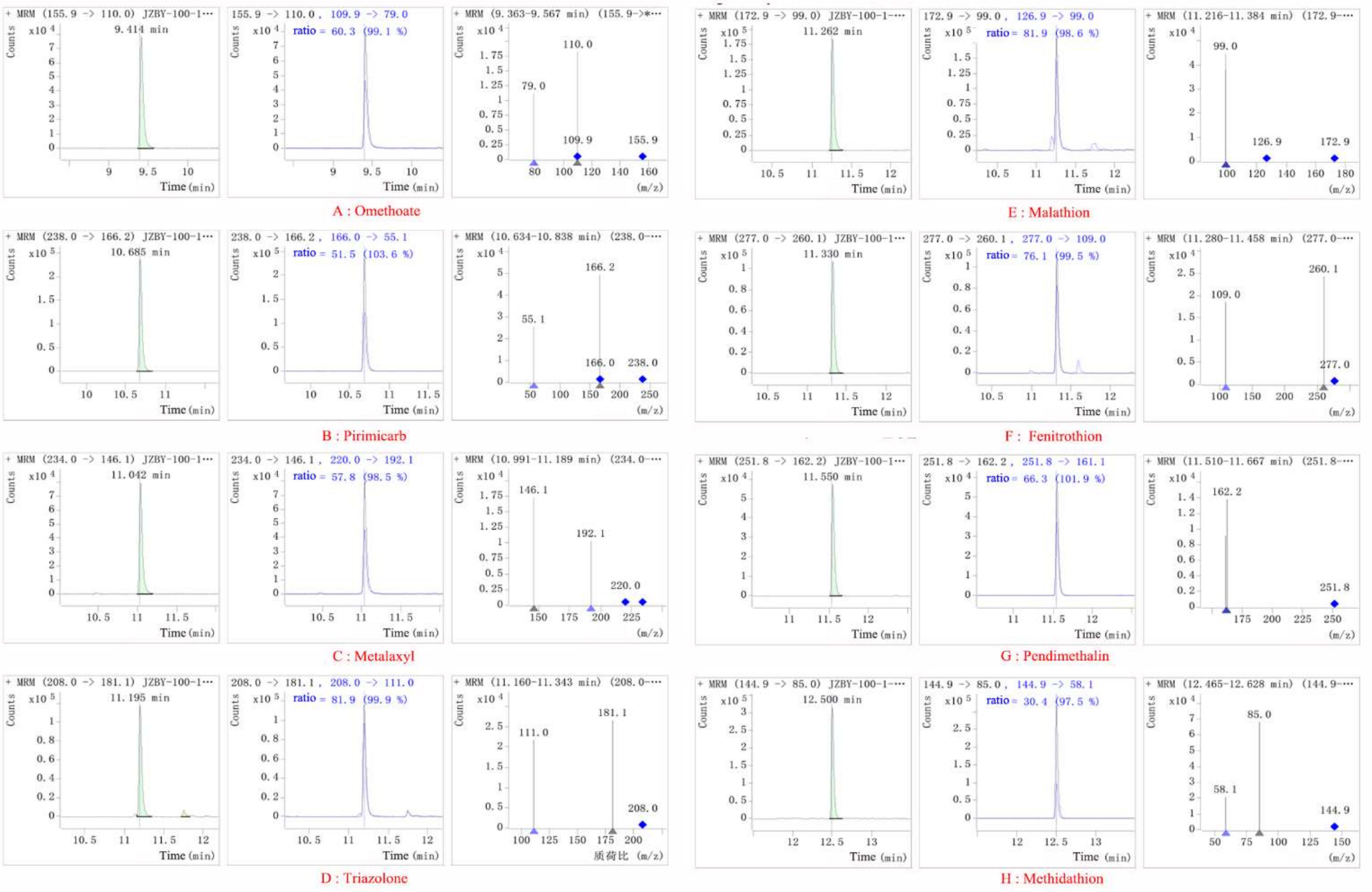

Figure S3 The total ion Chromatogram in MRM Mode, Qualitative and Quantitative Chromatogram of ion pair and Mass Spectrogram (A, Omethoate; B, Pirimicarb, C,.Metalaxyl; D, Triazolone; E, Malathion; F, Fenitrothion; G, Pendimethalin; H, 194 Methidathion).

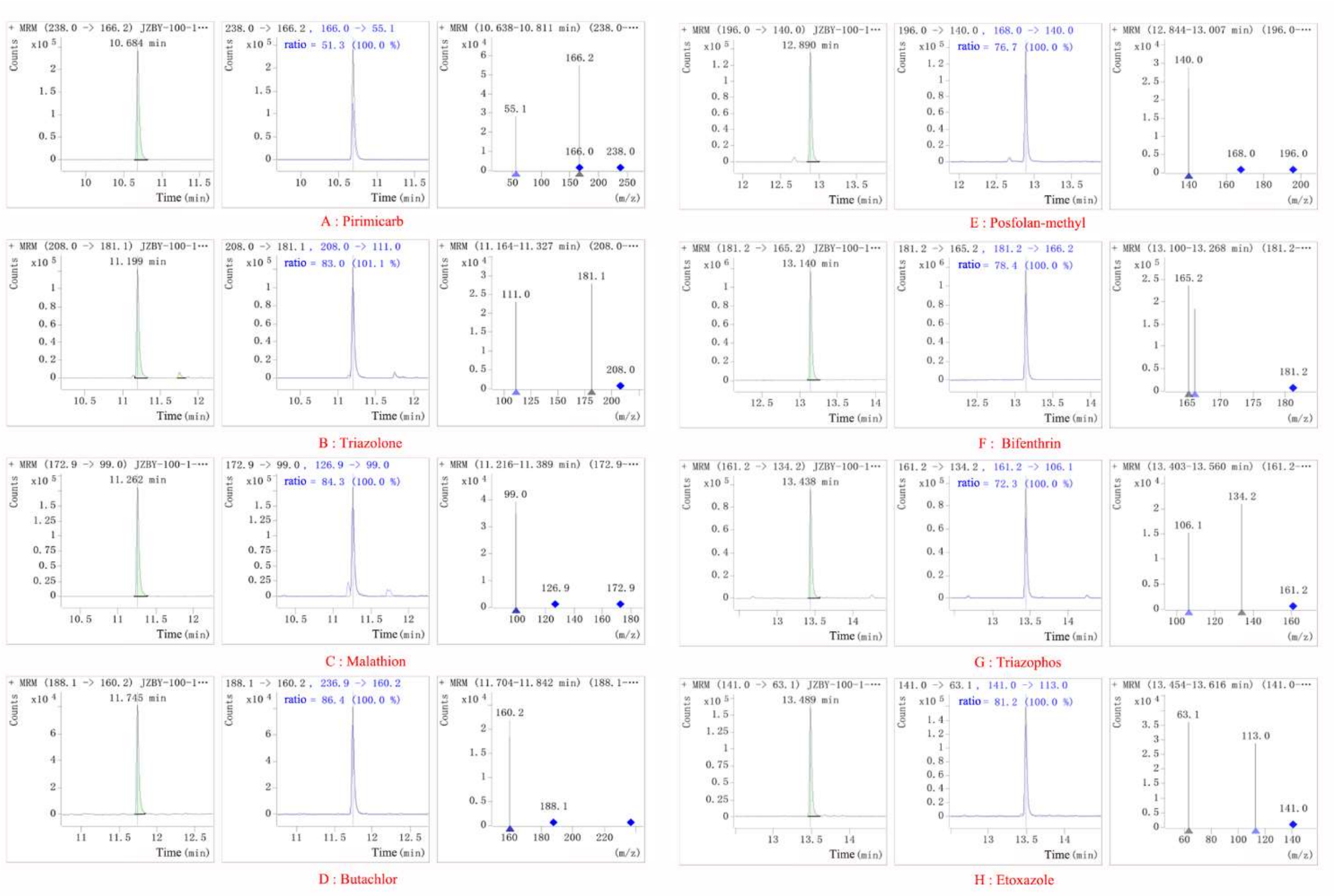

Figure S4 The total ion Chromatogram in MRM Mode, Qualitative and Quantitative Chromatogram of ion pair and Mass Spectrogram (A, Pirimicarb; B, Triazolone, C, Malathion; D, Butachlor; E, Posfolan-methyl; F, Bifenthrin; G, Triazophos; H, Etoxazole).
